# The study on spatial distribution of water ecological environment carrying capacity during extreme drought conditions

**DOI:** 10.1038/s41598-024-62856-9

**Published:** 2024-05-25

**Authors:** Yang Zhou, Yingying Gui, Qiang Zhou, Li Li, Miaomiao Chen, Yuling Liu

**Affiliations:** 1 School of Architecture and Civil Engineering, Chengdu University, Chengdu, 610106 Sichuan China; 2 Key Laboratory of Pattern Recognition and Intelligent Information Processing of Sichuan, Chengdu University, Chengdu, 610106 China; 3 State Key Laboratory of Eco-hydraulics in Northwest Arid Region, Xi’an University of Technology, Xi’an, 710048 Shanxi China; 4 The Meat Processing Key Laboratory of Sichuan Province Chengdu University, Chengdu, China; 5 College of Computer Science, Chengdu University, Chengdu, 610106 Sichuan China; 6 Sichuan Academy of Agricultural Machinery Sciences, Chengdu, China; 7 Xi’an University of Technology, Xi’an, 710048 Shanxi China

**Keywords:** Water ecological environment-carrying capacity, Wei River, Water environment indicator, Ecology, Environmental sciences, Hydrology

## Abstract

Due to global warming and the disturbance of the interannual variability of precipitation, the frequency of extreme drought events has increased. The impact of global climate change on water resources is becoming increasingly apparent, then it is particularly necessary to explore the carrying capacity of water ecological environment under extreme drought conditions, which can guarantee the ecological water security in river basins. This study takes the Guanzhong area of the Wei River Basin as an example, calculating the water environment carrying capacity of 40 areas in the Weihe Guanzhong area in different levels of years under extreme drought conditions by comprehensive evaluation model of carrying capacity and using geographic information system GIS to display the spatial distribution of water environment carrying capacity in 40 regions. According to the results of the spatial distribution of water environmental bearing capacity, four different schemes are designed to improve the bearing capacity. The first plan reduces the industrial water consumption and irrigation quota by 5%, the second plan increases the industrial water and sewage treatment rate on this basis. the third plan further improves the development and utilization rate of surface and groundwater, and the fourth plan, on the basis of the first three plans, supplies 600 million cubic meters of industrial and agricultural water to Guanzhong region. Through comparative analysis, without taking any measures, under the extreme drought conditions, the water environment carrying capacity of the 40 areas in Guanzhong is all in an unbearable state. Overall, plan 4 has the most significant improvement in the water environment-carrying capacity, especially the Dong zhuang Reservoir of the Jing River which has played a very important role in enhancing the water ecological environment carrying capacity of the downstream water of the Wei River.

## Preface

Extreme climate events are one of the most serious natural disasters to human society^1,1^. Due to global warming and the disturbance of the interannual variability of precipitation, the frequency of extreme drought events has increased. Global warming will accelerate the hydrological cycle process, causing changes in the amount of water resources and their spatial distribution, which will make the problem of water shortage more prominent in China, and the problem of aquatic ecological environment may also deteriorate further. At the same time, the unreasonable development and utilization of water resources has caused a bad water ecological environment. The impact of global climate change on water resources is becoming increasingly apparent. It is particularly necessary to further explore the water resource allocation and ecological environment carrying capacity (ENCC) under extreme drought conditions to guarantee the ecological water security and water supply security of the basin^3,4^. ENCC can be defined as the maximum human activity level that the regional water environment can support in a certain period of time -without adverse effects on the local water environment. Many scholars^5^ at home and abroad have conducted many research on the water environment-carrying capacity of different river basins or regions, which has laid a solid foundation for the study of regional socio-economic sustainable development and environmental planning. This has, to a certain extent, promoted the research and development of water environment-carrying capacity.

In recent decades, there have been many methods for evaluating regional water environmental-carrying capacity, such as system dynamics model^6–9^, fuzzy comprehension evaluation method^10,11^, MOP (multi-objective-programming)^12–15^, PCA (principal component analysis)^16,17^, ecological footprint analysis^18,19^, data envelopment analysis^20^. Each evaluation method has its advantages and disadvantages. Ren et al.^21^. Integrated fuzzy comprehension evaluation method (FCE), (AHP) and (CM) methods to evaluate water resources carrying capacity. System dynamics model^22^ considers the dynamic feedback relationship among the internal factors of the system in the dynamic quantitative evaluation. However, the establishment of this model is more complicated, and the dynamic feedback relationship needs to be sorted out. Fuzzy comprehensive evaluation method^11^ can deal with fuzzy evaluation objects through precise digital means, but the calculation process is complicated, and the determination of the index weight vector is subjective. MOP^23,24^ integrates the relationship between regional socio-economic and natural resources, and considers the development goals of different periods when making decisions. However, due to the lack of appropriate optimization methods, the application of this method is limited. PCA^25^ can eliminate the correlation between evaluation indicators and reduce the workload of indicator selection. Besides, comprehensive evaluation method^26^ has a certain degree of ambiguity and cannot express the results clearly. Although Bu et al.^27^ combined system dynamics, analytic hierarchy process (AHP) and MIKE11 model to predict WECC in Changzhou, they ignored the randomness and uncertainty in the prediction process, which may reduce the accuracy and reliability of the prediction. Wu et al.^28^ used fuzzy comprehension evaluation method to predict the water resource-carrying capacity in the Hei River. But this method was mainly used for evaluation rather than prediction, which reduced the effectiveness and applicability of the prediction. Taking Wuhan urban agglomeration in the middle reaches of the Yangtze River as an example, based on the improved ecological footprint model, the per capita ecological footprint and ecological-carrying capacity of Wuhan metropolitan area in three periods were calculated. In the evaluation process, Xiao comprehensively took biophysical factors into consideration and the ecological footprint index is highly referenceable. However, this method is over a single, and does not consider the impact of technology, management and other factors on it. Taking Xiamen City as a case study , Sun et al. conducted an energy analysis to calculate the energy flow within the city. Three sustainability indicators—sustainable development index, sustainable development energy index, and urban health level energy index—were selected to comprehensively evaluate its ecological sustainability. This evaluation used a consistent energy standard to facilitate quantitative and comparative analysis. Whereas, the method is too simplified and the evaluation results are not objective enough.

Above research methods are complex and diverse, which has achieved abundantly on water environment-carrying capacity. Therefore, based on previous research methods, the present work used the mathematical model of single-index water environment indicator and the improved analytic hierarchy process to study the water environment-carrying capacity. This method is simple and easy to analyze which opens up a new idea for the research method of carrying-capacity. In Guanzhong area of Wei River, there are significant differences in the development of social and economic, water exploitation and water environment problems among different regions. In order to further understand the carrying-capacity of each region and provide a reliable basis for decision-making, the simple water environment-carrying capacity evaluation model and GIS system developed in this study can effectively calculate and predict annual water environment-carrying capacity at various levels in the future. Moreover, they also can express the calculation results in spatial display. And the evaluation conclusion is consistent with the results caused by serious pollution in this area by the application in Guanzhong area of Weihe River Basin.

Firstly, according to the current situation of water environment and water resources utilization in Guan zhong area of Wei River, a suitable evaluation index system was established after index screening. A total of 12 representative key indicators were selected in this study. Secondly, the study analyzed and calculated the mathematical model of power function index carrying degree, and obtained 12 single-index carrying degree respectively. Then, the water environment-carrying capacity of Guan zhong area of Wei River was determined by water environment indicator and index weight. The study counted the water environment-carrying capacity of 40 regions in Guan zhong section of Wei River in different years. According to development of the regional economy of Wei River, four different schemes were designed and calculated the spatial distribution of the water environment-carrying capacity of the Guanzhong area of the Wei River Basin based the four schemes. This study explored ways of scientific and reasonable exploitation of water resources via the water environment-carrying capacity research, so as to achieved the coordination between social and economic development and the water environment-carrying capacity. Furthermore, the research has practical significance for the healthy development of economy and the improvement of people's quality of life in Guanzhong area of Weihe River.

## Materials and methods

### Establishment of water environment-carrying capacity evaluation index system

Reasonable screening of indicators is the premise of quantitative research on water environment-carrying capacity, and the index screening is directly related to the accuracy of the results. In general, the study of water environmental-carrying capacity needs to select several major indicators such as the index of social and economic development, environmental pollution and water resources utilization. Therefore, it is necessary to classify the indicators in detail again, and construct an index system to express various contributory factor in an accurate and reasonable way according to the actual situation of the Guanzhong area of the Weihe River. The selected indexes should follow the principles of scientific, operability and feasibility. The river's ecological base flow ensures a continuous water supply throughout the year, serving as a crucial factor in supporting the river ecosystem and playing an indispensable role in maintaining its healthy development. This study considered the ecological base flow under extreme drought conditions as a key indicator. It analyzed key indicators affecting water environment and resource utilization, and established a water environment evaluation index tailored to the actual situation in the Guanzhong area of the Wei River with 12 indicators totally,as shown in (Table 1).

**Table 1:** Evaluation index system of carrying capacity in Guanzhong region.

Target layer	Index layer	Meaning
Water ecological environment-carrying capacity	GDP per capita (RMB/person)	Ratio of GDP to total population
	Per capita net income of rural residents (RMB/person)	Ratio of net income of rural residents to rural population
	Water efficiency (%)	Ratio of basin or regional water consumption to total water resources
	Water resources per capita	Ratio of total water resources to population in basin or region
	Industrial water reuse rate (%)^28^	Ratio of industrial repeated water consumption to total industrial water consumption
	Centralized treatment rate of sewage	The proportion of domestic sewage treated by domestic sewage treatment plants to the total domestic sewage discharge
	COD environmental capacity loading rate (%)	Ratio of COD environmental capacity to total COD emission
	Dilution-ratio	Ratio of sewage discharge to river runoff
	Cultivated land irrigation rate^29^	Ratio of effective irrigated area to cultivated land
	Ecological base flow^30,31^	Minimum amount of water to maintain an ecosystem from destruction
	Vegetation coverage (%)^32,33^	Ratio of total area of grassland to total area of land
	Lose rate of water and soil (%)	Ratio of total land loss area to total land area

### Calculation of water ecological environment-carrying capacity

**Figure 1:**
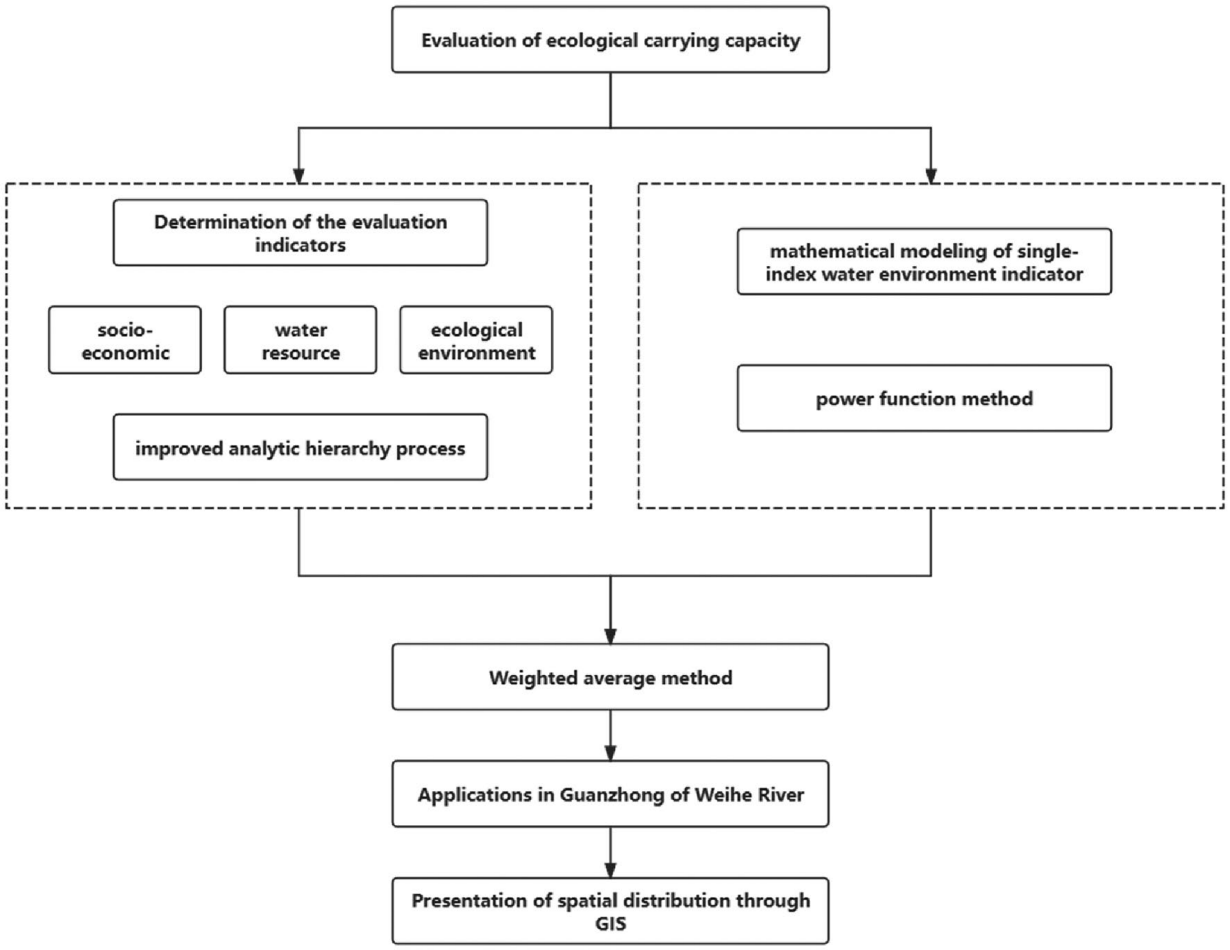
Flowchart of the integrated model framework.

Water ecological environment-carrying capacity was calculated by the weighted average method, which is to transform the statistical indicators of multiple directions affecting the carrying capacity into dimensionless relative evaluation values, and then evaluates regional water environment-carrying capacity comprehensively Firstly, through the screening of water environment indicators, 12 carrying degree evaluation indicators were calculated by using the single-index power function bearing degree calculation model. The single-index bearing degree represents the ratio of a single index value to the critical state index value. Secondly, the bearing degree of a single index was brought into weighted average evaluation model of the water environment-carrying capacity for calculation, and finally the carrying capacity of the regional water environment was obtained. Flowchart of the integrated model framework is shown in (Fig. 1).

Based on the definition of water ecological environment-carrying capacity, the improved analytic hierarchy process combined with the single-index power function was used to calculate the weighted average to water environment-carrying capacity. The comprehensive evaluation model of water environment-carrying capacity is as follows^34–38^:

1 $${\rm E} = \sum\limits_{i = 1}^{n} {\omega_{i} {\rm E}_{i} }$$

$E$ is water environment-carrying capacity, ${\omega }_{i}$ is weight of each index, ${E}_{i}$ is single-index carrying capacity.

#### 1 Mathematical model of single index carrying degree

This study calculated the carrying capacity of 12 indicators by mathematical model. The mathematical model established was to figure out the dimensionless bearing degree of each index, so that the bearing degree of the index is between 0 and 1. Where, 0 is the worst value of the index bearing degree, and 1 is the optimal value of the index bearing degree. Therefore, the pass value of the index carrying capacity is 0.6. Whether bigger is better or smaller is better, the bearing degree of index is between the optimal and worst values, and the function is monotonic. The functions that satisfy the requirements are power function and logarithmic function with exponents less than 1. This paper selects the power function as follows:

2 $$y = a + bx^{\frac{1}{2}}$$

y is the bearing degree of a single-index, x is the single indicator, a and b are the parameters in the model respectively. The selection of the values a and b refers to the "Basic Standards for the Well-off Living Standards of the National People" in China^39^, the internationally recognized index values, and the actual situation of China's economic development and social population. The standard values of some variables refer to the environmental standard values stipulated by the State Environmental Protection Administration on the construction indicators of ecological cities and counties^40^. Finally, the optimal and worst values of each indicator were determined. And the parameters of the bearing degree model of each index were reasonably obtained and calculated. The specific determination method is illustrated by the pollution diameter ratio in the water pollution index, which is an important evaluation index to measure the environmental quality of a river basin, and is an indicator that the smaller the better. The specific method was illustrated by the dilution-ratio of the water pollution index, which is a significant evaluation index to measure the environmental quality of a basin, and it is a smaller the better index. According to the above standard values, when the dilution-ratio took 1 and 0.04 as the worst and pass value respectively, which were substituted into Eq. (2) to obtained that a was 0.75 and b was 0.75. The calculation model of dilution-ratio was $y=0.75+0.75{x}^\frac{1}{2}$, when the bearing capacity was 1, the dilution-ratio was not 0, but 0.09, which was because the self-purification ability. It made sense to show that this model was practical and the remaining indicators could be determined in a similar way.

#### Determination of indicator weights

In the index system of water environment-carrying capacity, the weight of 12 indexes were determined by the improved analytic hierarchy process^41,42^, which adopted the 0, 1, 2 scale method as decision makers can accept and judge easily, as shown in (Table 2). The improved analytic hierarchy process did not need to construct a judgment matrix, nor did it need to perform a consistent test. The method was intuitive, simple, with order retention and without requirement for the number of indicators.

**Table 2:** Ratio of the importance of two adjacent indicators ($${r}_{k})$$.

Weight value	Comparative degree
1.0	Index sk-1 and sk are equally important
1.2	Index sk-1 and sk are slightly important
1.4	Index sk-1 and sk are obviously important
1.6	Index sk-1 and sk are strongly important
1.8	Index sk-1 and sk are extremely important

Supposing that decision makers rank n evaluation indicators in order of importance as (${s}_{1}>{s}_{2}>\cdots {s}_{n}$)

3 $$r_{k} = \frac{{\omega_{k - 1} }}{{\omega_{k} }} \, \left( {k = 2,3, \cdots ,n} \right)$$

${r}_{k}$ is ratio of the importance of two adjacent indicators, ${\omega }_{k}$ (k = 2,3,…n) and ${\omega }_{k-1}$ represent the k_th_ and (k-1)_th_ indicator weight, respectively.

4 $$\omega_{n} = \frac{1}{{1 + \sum\limits_{k = 2}^{{\text{n}}} {\prod\limits_{i = k}^{n} {r_{i} } } }} \, \omega_{k - 1} = r_{k} \omega_{k} \left( {k = n,n - 1, \cdots 2} \right)$$

### Comparative analysis and verification of water environment-carrying capacity evaluation system model

In order to verify the rationality of the model, the weighted average and fuzzy comprehensive evaluation method were used to analyse and evaluate the water environment-carrying capacity in the Baoji of Wei River in the normal year. Fuzzy comprehensive evaluation method was a comprehensive evaluation method based on fuzzy mathematics, which used the Table 1 index system to compare and analyse the evaluation results with reference, as shown in (Table 3).

**Table 3:** Evaluation result for water environment carrying capacity calculated by different evaluation method in different years.

Year	2005	2015	2020	Optimal value of water environment -carrying capacity
Weighted average method	0.416	0.402	0.375	0.996
Ratio of evaluation value to optimal value	0.418	0.404	0.377	
Fuzzy comprehensive evaluation method	0.385	0.374	0.352	0.8
Ratio of evaluation value to optimal value	0.481	0.468	0.44	
Vector modulus method	0.0231	0.021	0.0207	0.063
Ratio of evaluation value to optimal value	0.367	0.333	0.329	

In Table 3, there were 3 different methods to calculate water environment-carrying capacity, which all showed the descent of the water environment-carrying capacity in Baoji within years. According to the optimal value of internationally recognized indicators, different evaluation methods were used to determine the optimal value of water environment-carrying capacity. The ratio of the evaluation value to the optimal value means that it is the evaluation value of a method that comparing optimal bearing capacity level. Compared with the fuzzy comprehensive evaluation method, the bearing capacity level of the weighted average method was close to that of reference. The bearing capacity level of the fuzzy evaluation method was larger. Fuzzy comprehensive evaluation method uses the membership degree to judge, and there is a value within the membership degree, and the standard in the non-membership degree is 0. The comprehensive evaluation results of such multiple indicators were too two-tiered, resulting in unreasonable evaluation results and greater defects in ambiguous evaluation methods and the defect of fuzzy evaluation method was relatively big. The results of reference were the blatant results of the Baoji in Weihe River and were considered to be correct and reasonable, indicating that the results calculated by the weighted average are also reasonable. Moreover, this method has the characteristics of simple calculation, which is easy to promote and use.

### Different schemes of water ecological environment-carrying capacity in Guanzhong of wei river

Four different schemes are designed to improve the bearing capacity. The first plan reduces the industrial water consumption and irrigation quota by 5%, the second plan increases the industrial water and sewage treatment rate on this basis, the third plan further improves the development and utilization rate of surface and groundwater, and the fourth plan, on the basis of the first three plans, supplies 600 million cubic meters of industrial and agricultural water to Guanzhong region.”

## Application

### Study area

**Figure 2:**
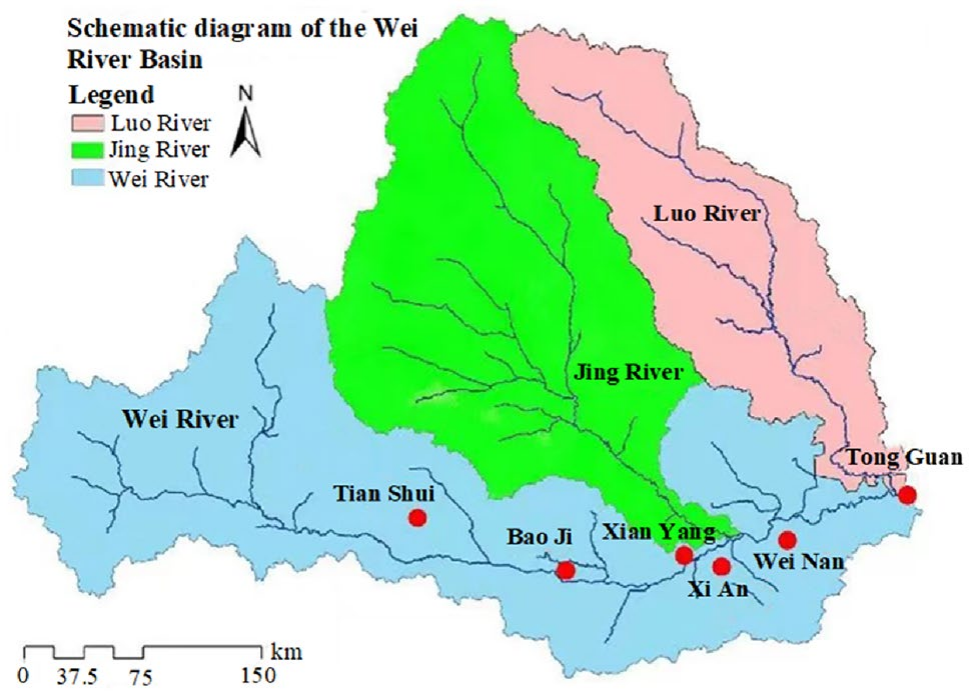
Schematic diagram of the Wei River Basin (Map processed by Authors using: ArcGIS 10. software:https://www.tianditu.gov.cn).

Wei River is a primary tributary of the Yellow River, originating in Weiyuan county, Gansu Province, as shown in the Fig. 2. After flowing through Weiyuan, Wushan, Tianshui, and Gangu in Gansu Province, the Wei River enters Shaanxi Province at Fenggeling. It then crosses Baoji, Xi'an, Xianyang, Weinan, Yangling, and other urban areas before ultimately joining the Yellow River at the port of Tongguan. The river covers a total area of approximately 13.5 × 10^4^ km^2^ and spans a total length of 818 km. The Guanzhong section of the Weihe River crosses the east and west of Shaanxi Province and mainly flows through the Guanzhong region, the economic centre of Shaanxi Province. Guanzhong includes 5 cities and 1 district and 47 counties which are all or part counties (cities, districts), including Baoji, Yangling, Xianyang, Xi'an and Weinan. The total population, arable land, irrigated area and GDP of the Weihe Guanzhong region account for 64%, 56%, 72% and 80% of Shaanxi Province respectively. As the Wei River is a seasonal river, temporal and spatial distribution of rainfall in the basin is extremely uneven. Historically, floods and droughts have occurred frequently, even each drought has lasted for a long time, causing very serious consequences. According to the Atlas of Drought and Flood Distribution in China in the Last 500 Years, the Weihe River was also a continuous severe drought in the Ming Chongzhen 5–15 years and the Republic of China 11–21 years. In the 10 years of Ming Chongzhen 5–5 years, there was a drought that lasted for 9 years. For 12 years of drought in the Republic of China 11–21 years, 5 years drought was continuous from 1928 to 1932. Moreover, Henan, Gansu and Ningxia, provinces around the Wei River, have also experienced different degrees of drought. According to the rainfall records, the average annual rainfall of Xi'an was 327.87 mm, which was 57% of the annual average from 1928 to 1932. The year with the lowest rainfall was 1928, and the rainfall was 42% of the multi-year average, indicating the continuous drought in the Guanzhong region during this period.

### Evaluation indicator system weight in the study area

In the index system of water environment-carrying capacity, the weight of 12 indexes were determined by the improved analytic hierarchy process^41,42^, which adopted the 0, 1, 2 scale method as decision makers can accept and judge easily, The weight of each index calculated is shown in (Table 4).

**Table 4:** Index weight calculation results.

Index meaning	Weight	Index weight
GDP per capita	${\omega }_{1}$	0.043
Per capita net income of rural residents	${\omega }_{2}$	0.097
Water efficiency	${\omega }_{3}$	0.077
Water resources per capita	${\omega }_{4}$	0.043
Dilution-ratio	${\omega }_{5}$	0.118
Industrial wastewater treatment rate	${\omega }_{6}$	0.047
Industrial water reuse rate	${\omega }_{7}$	0.052
Cultivated land irrigation rate	${\omega }_{8}$	0.037
COD environmental capacity loading rate	${\omega }_{9}$	0.105
Ecological base flow	${\omega }_{10}$	0.201
Vegetation coverage (%)	${\omega }_{11}$	0.044
Lose rate of water and soil	${\omega }_{11}$	0.136

### Results on water ecological environment-carrying capacity in Guanzhong of Wei river

The spatial scope of this study is the Guanzhong of Weihe River, and it is much significant among different regions in socio-economic development status, water exploitation, and water environment problems. In order to further understand the carrying capacity of each region and provide a reliable basis for decision-makers to make decisions, this research collected relevant data on socio-economic and population, water resources, water environmental pollution and discharge within ecological and environmental protection in the Guanzhong area of Weihe. According to the improved analysis hierarchy process, the weight calculation of each index and single index carrying degree is determined. Subsequently, the water environment-carrying capacity of the Guanzhong area of the Weihe River was calculated using Eq. (1), and the spatial distribution of carrying capacity across 40 regions was displayed using GIS.

**Figure 3:**
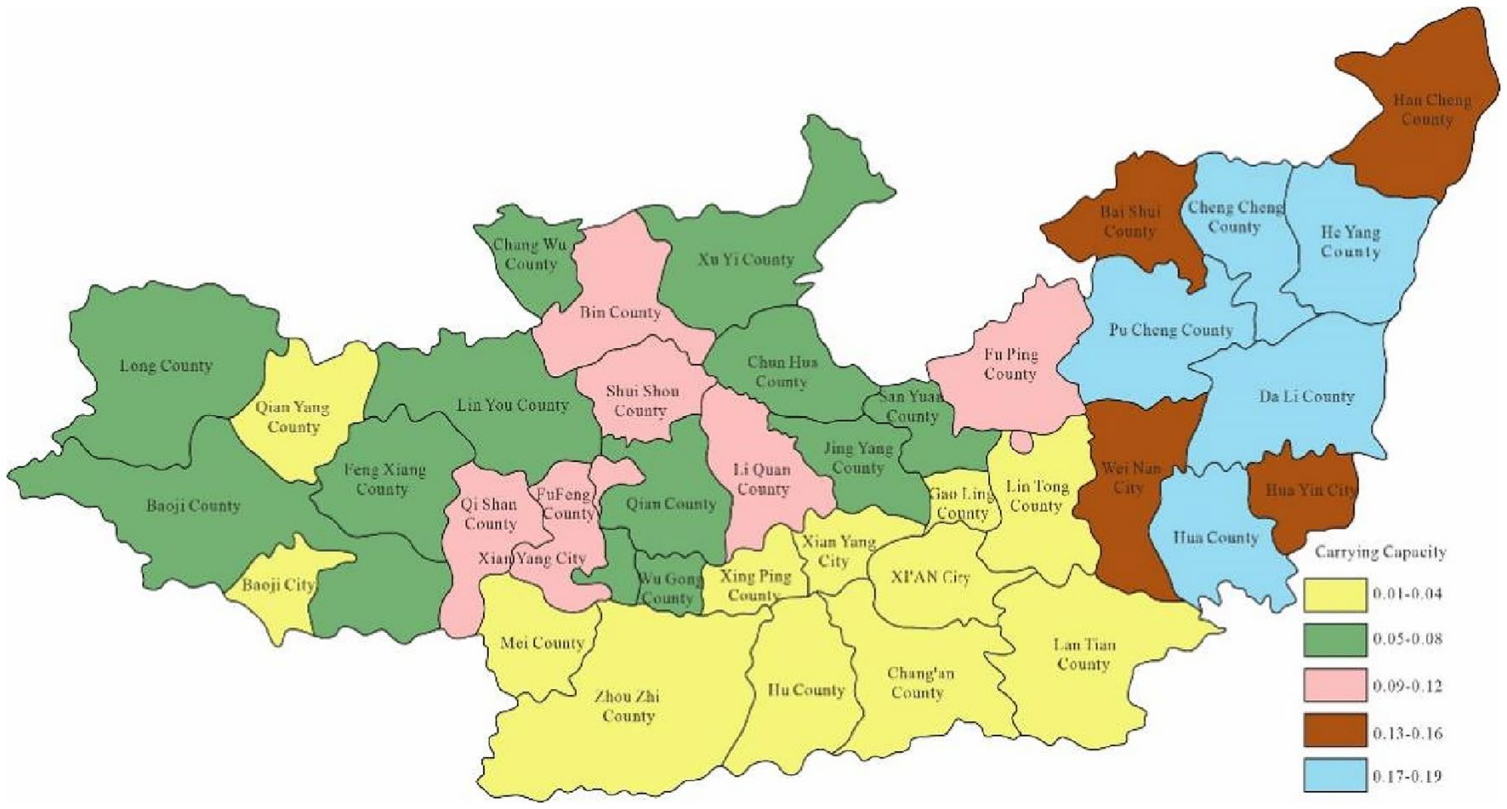
Space distribution of water environmental carrying capacity in2023 (Map processed by Authors using: ArcGIS 10. software:https://www.tianditu.gov.cn).

Through the constructed regional carrying capacity evaluation model, the study obtained the water environment-carrying capacity of 40 areas in the Guanzhong section of the Weihe River within years. Moreover, the study took 2023 as the current level year which hypothesised the carrying capacity in 2028 and 2030 respectively. Figure 3 is the spatial distribution map of the calculation results of water environment-carrying capacity in Guanzhong of Weihe River in 2023.

**Figure 4:**
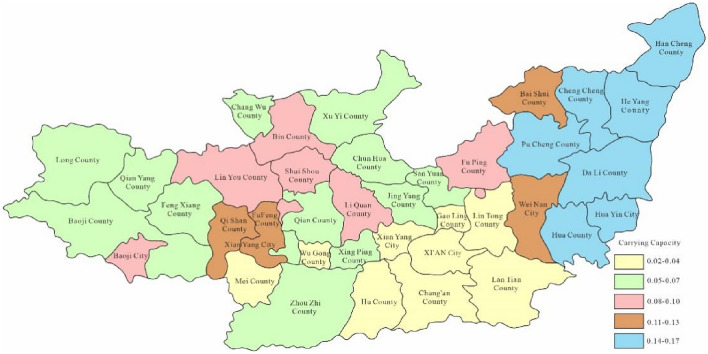
Space distribution of water environmental carrying capacity in scheme 1 in 2028 (Map processed by Authors using: ArcGIS 10. software:https://www.tianditu.gov.cn).

**Figure 5:**
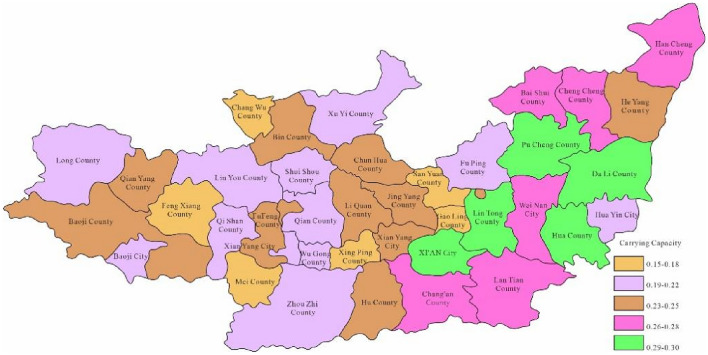
Space distribution of water environmental carrying capacity in scheme 2 in 2028 (Map processed by Authors using: ArcGIS 10. software:https://www.tianditu.gov.cn).

**Figure 6:**
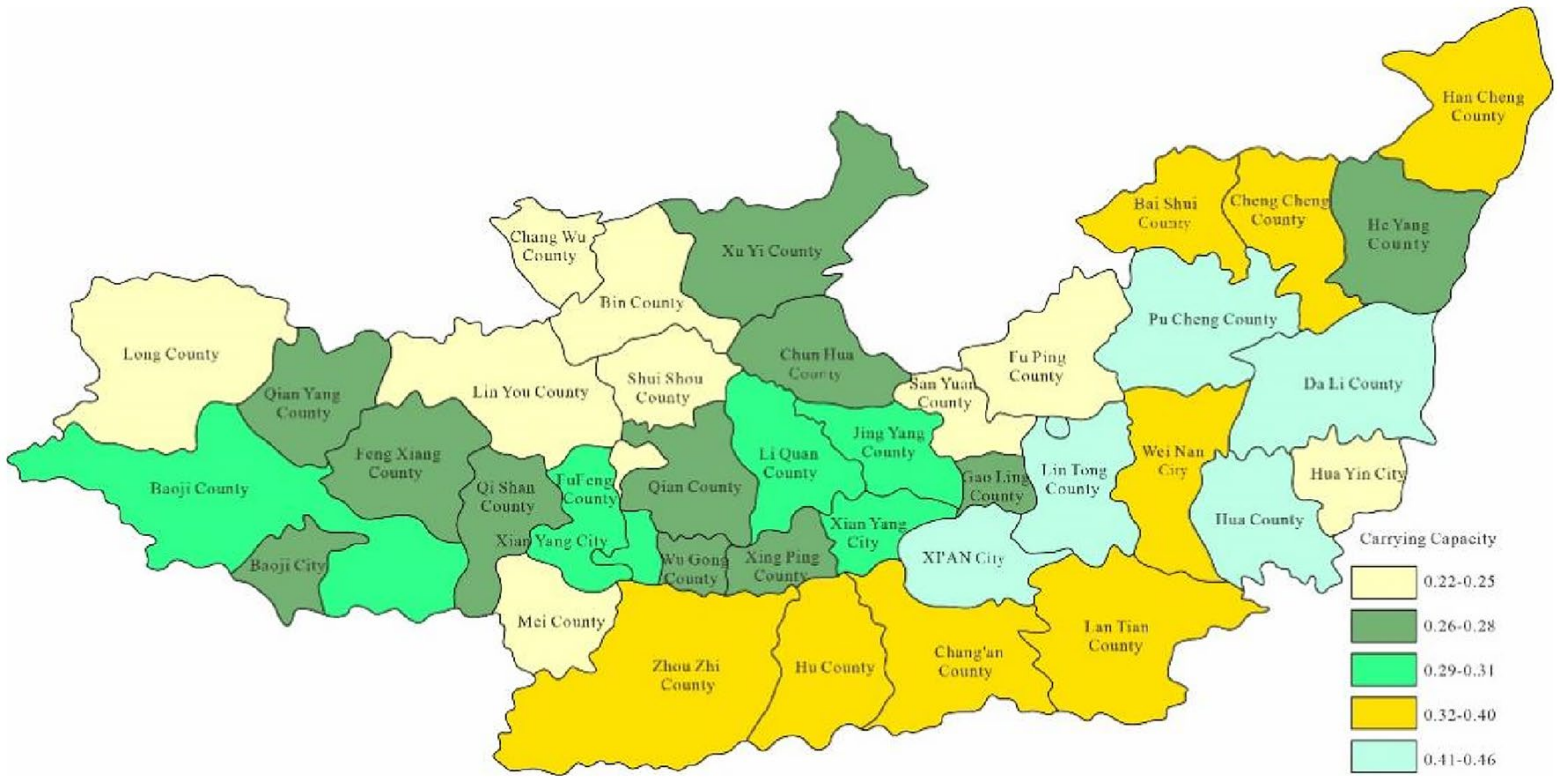
Space distribution of water environmental carrying capacity in scheme 3 in 2028 (Map processed by Authors using: ArcGIS 10. software:https://www.tianditu.gov.cn).

**Figure 7:**
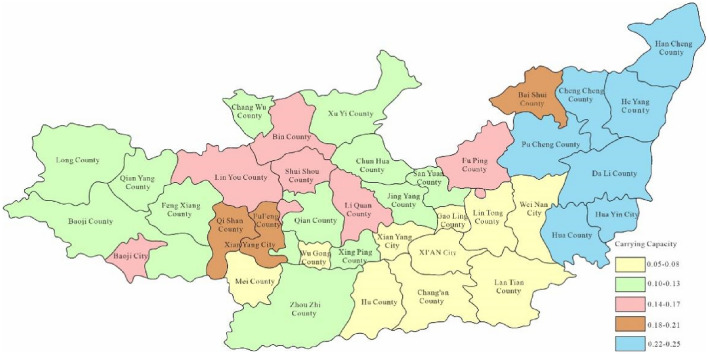
Space distribution of water environmental carrying capacity in scheme 1 in 2030 (Map processed by Authors using: ArcGIS 10. software:https://www.tianditu.gov.cn).

**Figure 8:**
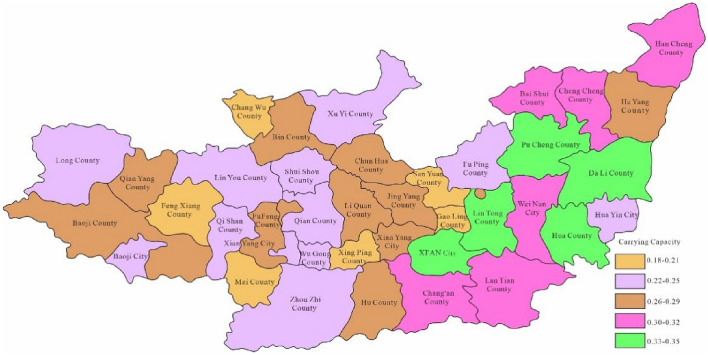
Space distribution of water environmental carrying capacity in scheme 2 in 2030 (Map processed by Authors using: ArcGIS 10. software:https://www.tianditu.gov.cn).

**Figure 9:**
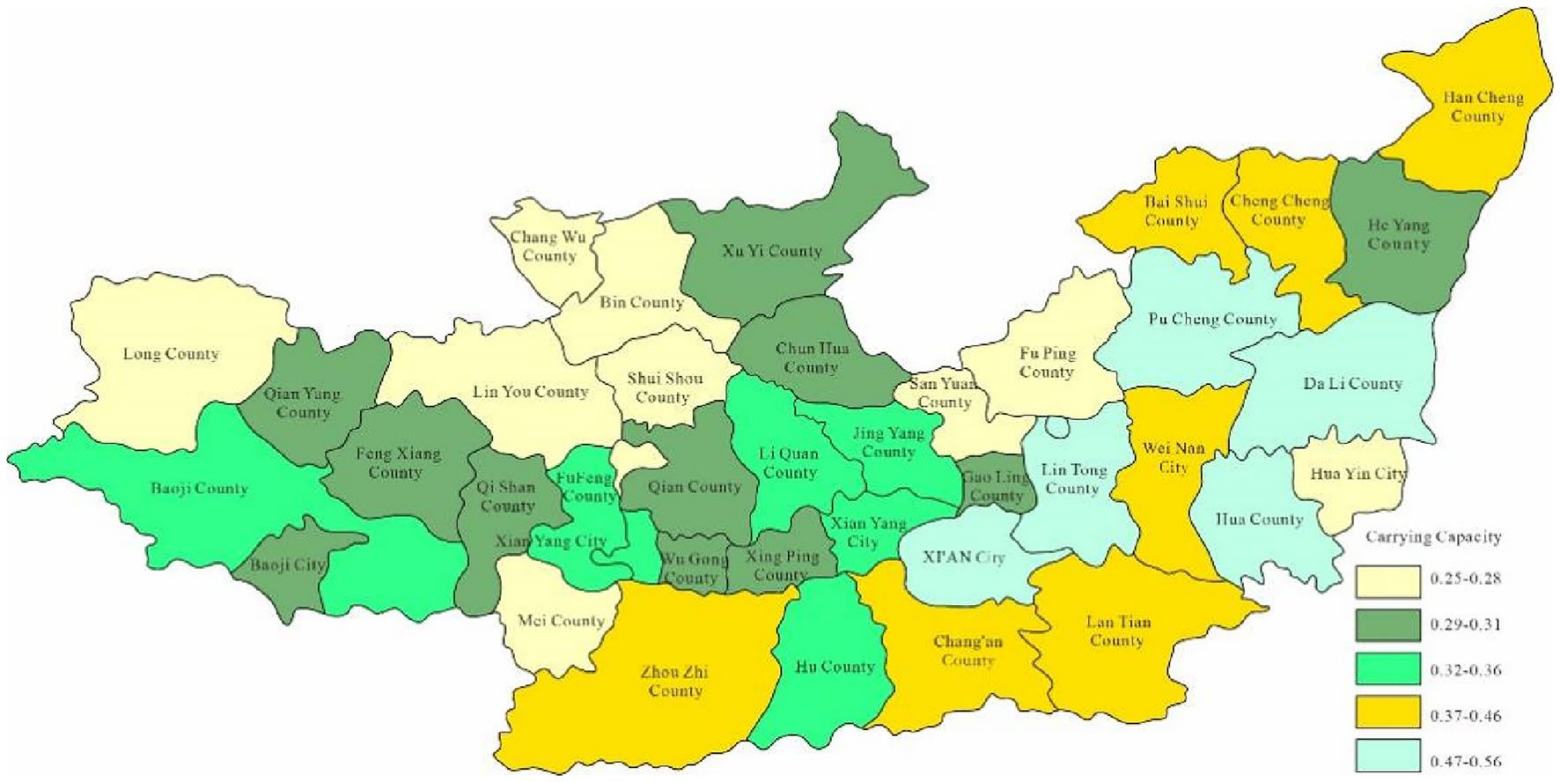
Space distribution of water environmental carrying capacity in scheme 3 in 2030 (Map processed by Authors using: ArcGIS 10. software:https://www.tianditu.gov.cn).

**Figure 10:**
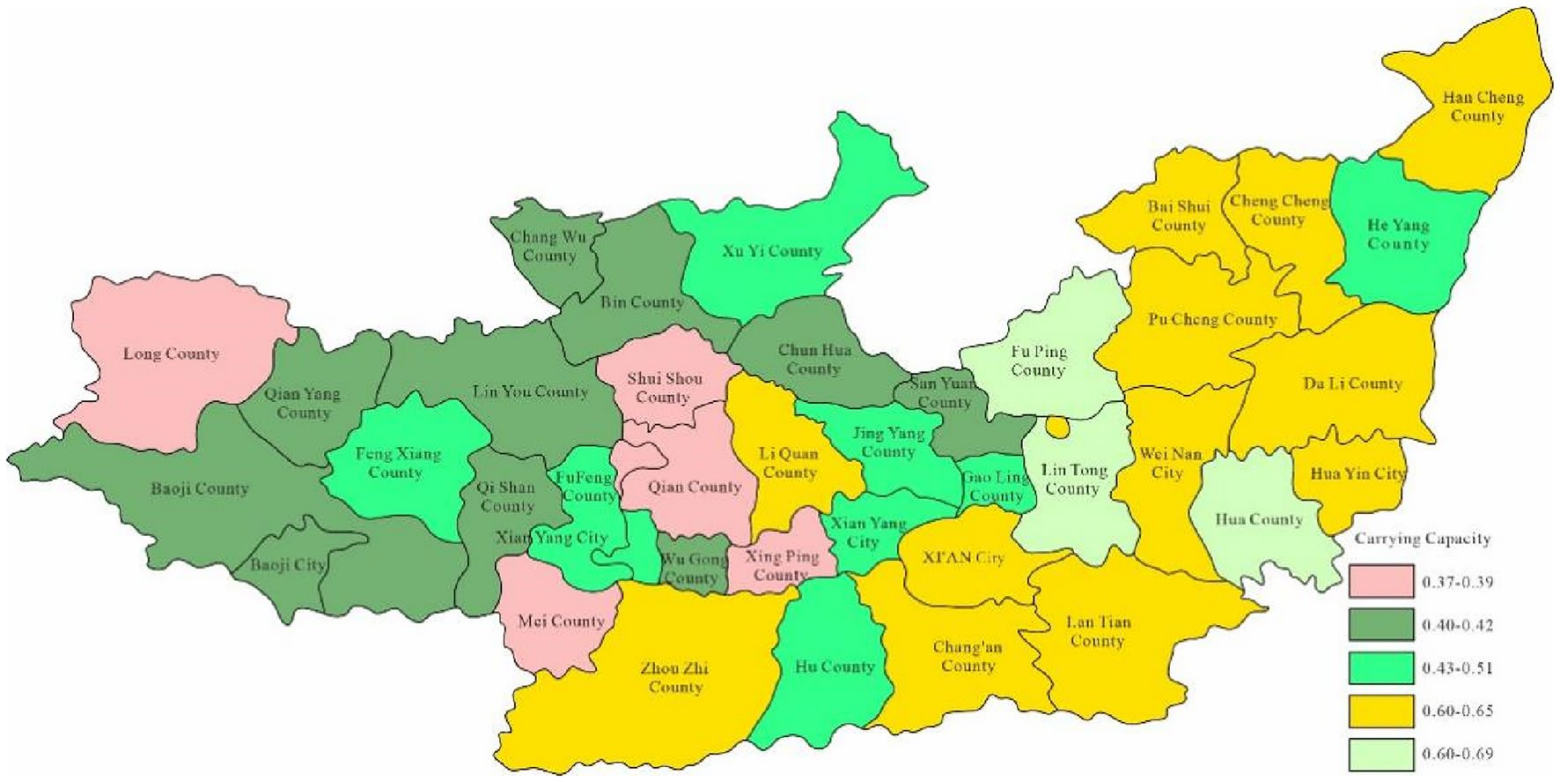
Space distribution of water environmental carrying capacity in scheme 4 in 2030 (Map processed by Authors using: ArcGIS 10. software:https://www.tianditu.gov.cn).

Through Fig. 3, the water environment-carrying capacity of the Guanzhong area of the Weihe River Basin was between 0.01 and 0.19 which was unbearable. This was mainly due to the water environment problems such as water shortage and serious water pollution caused by extreme drought events in the Weihe River that made the water environment-carrying capacity in Baoji City and Xi'an City relatively low. Since the water environment-carrying capacity reflects the natural and social attributes of the water resources system, its size is more closely related to the ability of regional pollution control. To enhance water environment-carrying capacity, this study designed water environment-carrying capacity under different scenarios. Schemes 1, 2 and 3 will be adopted in 2028, as to schemes 1, 2, 3 and 4 will be adopted in 2030. As was shown in Figs. 4, 5, 6, 7, 8, 9, 10, it was water environment-carrying capacity spatial distribution in Guanzhong of Weihe River in 2028 and 2030 respectively.

It can be seen from Figs. 4, 5 and 6 that the water environment-carrying capacity of each region under Scheme 1 in 2028 was at the non-carrying level, which has been improved under Scheme 2. The carrying capacity of most areas in Xi'an was at the weak carrying level where other areas were still at the non-carrying level and which showed that the treatment of Xi'an was still effective. Due to the implementation of water diversion project, the bearing capacity of some areas in Xi'an and Baoji has been greatly improved under scheme 3.

It can be seen that the industrial and agricultural water-saving measures have improved the water environment-carrying capacity under the original scheme by Fig. 7, which was still at the level of non-carrying capacity in all regions. Figure 8 showed that the carrying capacity of each region was basically at a weak carrying level under scheme 2, indicating that the improvement of sewage treatment rate and industrial water reuse rate was still effective. Figure 9 showed that due to the implementation of the water diversion project, the bearing capacity of most areas has been greatly improved comparing with scheme 2. However, as the extreme water shortage under critical drought conditions, the bearing capacity was at the upper limit of weak bearing capacity, close to the basic bearing capacity level. It can be seen from Fig. 10 that the construction of Dongzhuang reservoir in Jing river would significantly improve the bearing capacity of Guanzhong area of Weihe River, especially most of the lower reaches of Weihe River, which was basically a good bearing level.

The ENCC values calculated by single index load capacity and improved hierarchical analysis are shown in Table 5. Without any action or under scenario 1, the ENCC remains at an unsustainable level. In addition, our forecasts for plan 2 in 2028 and plans 1 and 2 in 2030 also indicate weak carrying capacity levels. Although the data of Scheme 3 are improved compared with the previous two schemes, they are significantly different from the bearing capacity value predicted in Scheme 4 in 2030. On the whole, the four schemes have significantly improved the three regions, and the four schemes are more effective on the improvement of Xi'an.

**Table 5:** Environmental carrying capacity for three regions under different scenarios.

Ecological and environmental carrying capacity in 2023		Ecological and environmental carrying capacity in 2028			Ecological and environmental carrying capacity in 2030			
Scheme	without taking any measures	1	2	3	1	2	3	4
Baoji	0.02	0.08	0.19	0.26	0.14	0.22	0.29	0.4
Xianyang	0.01	0.02	0.23	0.29	0.18	0.26	0.32	0.43
Xi’an	0.01	0.03	0.29	0.41	0.05	0.34	0.47	0.62

### Sensitivity analysis

The sensitivity analysis is conducted using the Baoji section of the Weihe River as an example. Table 6 presents the variations in the ecological carrying capacity of the water environment in the Baoji section of the Weihe River when the annual increase rates of industrial water reuse are 2% and 2.5%, respectively. It is evident that even a modest increase of 0.5% in the reuse rate can significantly enhance the ecological carrying capacity of the Baoji section of the Weihe River. This demonstrates the strong sensitivity of the industrial water reuse rate indicator.

**Table 6:** Water ecological environment carrying capacity of Baoji section of Weihe River under different increasing rates of industrial water reuse.

Year	Carrying capacity in the Baoji section of Weihe River	
	Increase of 2% in industrial water reuse rate	Increase of 2.5% in industrial water reuse rate
2023	0.01	0.02
2028	0.261	0.274
2030	0.282	0.315

## Discussion

### Discussion on the water ecological environment-carrying capacity

According to the economic development of each region in the Weihe River, four different schemes were designed. The study has calculated the spatial distribution of water environment-carrying capacity in Guanzhong of Weihe River under four schemes by the powerful spatial display function of GIS. Under the condition of existing management and without any measures,

The water environment-carrying capacity of Guanzhong was in a non-bearing state by comparative analysis. Water environment has become a constraint factor for the sustainable development of economy and society in river basin. Although a single measure can improve the regional water environment-carrying capacity, the effect was not significant instead that the open source, throttling, pollution control and other measures can enhance the water environment-carrying capacity apparently. Dongzhuang reservoir played a very important role in improving the water environment-carrying capacity of the lower reaches of Weihe river.

### Limitations and future research directions

Due to the complexity of the ENCC system, there are still some limitations and deficiencies in this study. First, although many factors were involved in this study, the NECC assessment system con-structed here in is still a simplified model. Some factors have been simplified or ignored for many reasons. For example, due to the large area and the complex water system in this study, it is very difficult to collect continuous aquatic biological index data for many years.

Therefore, the biological diversity indicators have not been taken into consideration. In most of the existing studies, biological diversity indicators were not included in the NECC evaluation system. However, biological diversity indicators play an important role in NECC evaluation, which can reflect the biodiversity in aquatic ecological environment. The lack of these indicators will lead to the neglect of the needs of nature and environment, resulting in the deviation of the evaluation results of the NECC. This is what we should pay attention to in future research.

In future research, we should continuously improve the evaluation criterion of each evaluation index according to the actual situation, and gradually establish a meaningful and scientific NECC grade standard. Inter basin water transfer projects will greatly improve NECC.

## Data Availability

Data is provided within the manuscript.
